# Potential for heat production by retrofitting abandoned gas wells into geothermal wells

**DOI:** 10.1371/journal.pone.0220128

**Published:** 2019-08-06

**Authors:** Asif Mehmood, Jun Yao, Dongyan Fan, Kelvin Bongole, Junrong Liu, Xu Zhang

**Affiliations:** School of Petroleum Engineering, China University of Petroleum (East China), Qingdao, China; UNILAB Research Center for Chemical Reaction Engineering, CHINA

## Abstract

Using abandoned gas wells as geothermal resources for energy production is an effective way to extract geothermal energy from geological formations. These abandoned wells have the potential to significantly contribute in the rising global demand for energy without requiring the land disruption resulting from deep drilling or digging, processes necessary for energy extraction from geological formations via more traditional methods. In this paper, a method to extract geothermal energy from abandoned gas wells is proposed. The method offers an efficient, economical, and environmentally-conscious way to generate electricity. A mathematical model of a thermal and hydraulic coupling process is constructed, and a 3D numerical model is generated to study the process of geothermal energy extraction by retrofitting an abandoned gas reservoir into a geothermal reservoir. Using the model, heat extraction and fluid flow are analyzed over a period of 50 years. The heat production, electricity generation, and thermal recovery over the lifetime of the reservoir indicate that a commercially viable geothermal dual well system can produce geothermal energy effectively. Dual-well systems contain at least one injection well and one production well. They are composed of a two-way flow system in which the fluid flows into the reservoir via an injection well and returns from the production well having absorbed thermal energy from the surrounding rocks. Sensitivity analysis of the main parameters controlling the average outlet temperature of the fluid from the sedimentary geothermal system reveals that abandoned gas wells are a suitable source of geothermal energy. This energy can be harvested via a method whose use of reservoir fluids differs from that of the traditional method of closed-loop circulation via a borehole heat exchanger. Here, it is demonstrated that abandoned oil and gas fields can be repurposed to be geothermal energy sources that provide low-cost electricity and are economically sustainable.

## Introduction

Energy resource availability has a direct influence on the economic development of a country. Urbanization, industrialization, and population growth, especially in developing countries, have resulted in a global energy crisis. Conventional energy resources, such as oil and natural gas, are not only costly but finite. In addition, the exploitation of these resources harms the environment. For example, CO_2_ is a greenhouse gas that contributes to global warming, and SO_2_ and NO_2_ are air pollutants [1]. Most conventional energy resources are not only non-renewable, but their supply is rapidly decreasing. Therefore, researchers and policymakers are looking to replace these non-renewable resources with viable alternatives, such as solar, wind, or geothermal energy or biogas generation. Geothermal energy is the thermal energy stored from sub-surface. This heat is generated in the earth’s interior during various geological processes. The major contributor to continental heat flow is the decay of radioactive isotopes, which release large amounts of energy [2]. Magmatic processes and the mantle’s stored thermal energy also play a significant role in the overall geothermal energy supply. A small number of radio isotopes are present in the oceanic lithosphere, but compared to that of the mantle, their contribution is insignificant. Interestingly, as geothermal energy is generated deep in the earth by its “internal heat engine,” the amount of stored geothermal energy increases with increasing depth. At shallower depths, fluids transport this energy to the surface, and it is referred to as “hydro-geothermal energy.” This type of energy is present in tectonically active areas containing hot springs, volcanoes, and geysers. Hot dry rocks (HDR) are another geothermal energy source. In certain areas, enough hydro-geothermal energy is present to efficiently run turbines and generate electricity. With a suitable extraction system, geothermal energy in these areas is a more sustainable and cost-effective option for electricity generation that is less destructive to the environment than more traditional energy sources [3].

Geothermal power generating systems are used globally. However, to develop a commercially viable geothermal power generating system, one must consider many factors, i.e., available prospecting, drilling, and reservoir technologies, energy costs in the area, and resource durability [4]. From 42–95% of the total geothermal project cost, which can be mitigated by repurposing abandoned exploratory wells, is devoted to the drilling process [5]. Oil and gas wells can aid in the extraction of subsurface geothermal energy. The drilled borehole provides useful geological, geophysical, and geochemical information about the sub-surface reservoirs and allows direct access to the sub-surface heat energy [6, 7]. Globally, there are many “candidate wells” that can be used for geothermal energy extraction. They are more common in mature oilfields. Poor production of fossil fuels from a well leads to its abandonment. Water flooding results in high water-cut wells that lose their commercial value. Such types of wells can be used for geothermal energy exploitation [8]. A high bottom-hole temperature, reliable wellbore integrity, and large production capacity make a well viable candidate for geothermal energy extraction. Because geothermal energy has caught the attention of geologists and other professionals in the energy industry, there is interest in modifying existing wells. They understand that abandoned oil and gas wells can play a vital role in geothermal resource utilization.

Sliwa T (2014) [9] proposed utilizing abandoned oil and gas wells near urban areas by introducing the concept of a wellbore heat exchanger. Dijkshoorn L (2013) [10] designed a mathematical model for constructing a deep co-axial borehole heat exchanger to control the climate of buildings in Aachen, Germany. They concluded that the application of the model was not economically feasible due to an expensive inner pipe. Caulk R A (2017) [4] presented a mathematical model for reusing abandoned oil and gas wells for geothermal energy production and predicted that a 1000m deep well can achieve a production fluid temperature of higher than 40°C in a region with a temperature gradient of up to 7°C/100m. Kohl T (2002) [11] investigated the system performance of a deep borehole heat exchanger and proposed a numerical simulation method to understand the heat transport process taking place in wellbore heat exchangers. Kujawa T (2004) [12] constructed a computational model for acquiring geothermal energy from an existing abandoned well and also proposed insulating the inner pipe to prevent heat loss. Zhang L (2008) [13] proposed an enhanced geothermal reservoir system and analyzed the possibility of extracting energy from abandoned petroleum wells. Davis A P (2009) [14], Bu X (2012) [15], and Templeton J (2014) [16] investigated the sensitivity of the various parameters and the feasibility of geothermal energy extraction from abandoned petroleum wells to generate electricity. Nian Y L (2018) [17] evaluated the production of geothermal energy from abandoned oil and gas wells. Recently, Macenić M (2017) [18] studied the extraction of geothermal energy from a deep dry well via closed circulation and concluded that abandoned oil and gas wells are economical in a variety of industrial and commercial sectors.

The Indus Basin contains about 689 exploratory wells and 450 abandoned wells [19]. Data from these wells provide a large amount of geological information about subsurface lithology, geological structures, temperature, and reservoir porosity and permeability [20]. This information, along with the previously-drilled boreholes, can be utilized to develop economically feasible renewable geothermal energy in the Indus Basin [21]. A notable aspect of the research presented in this paper is its identification of a rift valley located in the Indus basin that is a potential source of geothermal energy. Consecutively, the research question discussed in this study is whether the life span of geothermal energy extracted from the reservoir will deliver the required outlet temperature, heat production and power generation. From an engineering point of view, the reuse of abandoned wells to obtain efficient thermal gradients at minimum cost is an achievement. Pakistan is an under-developed country, and the provision of power is of prime concern. This research can easily be applied to improve the lives of the people in Pakistan because it is based here. The aim of this study is to evaluate heat production from abandoned gas wells in the Indus Basin, Pakistan, with the goal of extracting geothermal energy for power generation. A mathematical model of thermal and hydraulic coupling processes is used to generate an accurate 3D numerical model for the representation of an abandoned gas reservoir, which provides suitable conditions of a geothermal reservoir. Heat transfer and fluid flow through the reservoir are analyzed over a period of 50 years. An abandoned well located in the Chiltan Formation is used to obtain key geological parameters.

### Geothermal resources of Pakistan

Most geothermal reservoirs in the northern areas of Pakistan possess a temperature higher than 150°C and have the potential to generate electricity [22, 23]. Below is a brief synopsis of the geothermal resource potential of Pakistan. High temperatures are found at most geothermal resources because they occur within tectonically active areas, such as at plate margins and centers of volcanic activity. In addition, fault movements cause friction and can release heat to the surroundings [19]. Pakistan lies within a tectonically active zone and has numerous hot springs, geysers, and mud volcanoes. The geothermal resources are characterized by their different temperature ranges, most of which are low to moderate. From geothermal resources, electricity can be generated at temperatures > 100°C [24, 25], and low-temperature geothermal resources can be used to heat greenhouses and for fishing, farming, and bathing [26]. In identifying geothermal energy resources to generate electricity, the goal is to locate high-heat-generating bodies at shallow depths, i.e., between 3–5 km. Locating and studying prospective geothermal resources (and petroleum exploration) relies upon geological, magnetic, gravitational, and seismic data.

#### Geothermal heat-flow patterns

The Earth’s surface temperature, determined from satellite measurements, and thermal gradient data, obtained by exploratory wells are used to detect the geothermal heat flow pattern in various regions of Pakistan [21].Satellite-recorded earth skin temperature data were acquired from NASA (National Aeronautics and Space Administration) [27]. About 22 years of processed data and a first-round local map, which shows the skin’s thermal gradient distribution for Pakistan, was generated by Stackhouse P (2006) [27]. The 22-year average temperature is 29–31°C in the study location. The crustal heat flow in this area is high because the area is a failed rift valley. In the southeastern part of Pakistan (Karachi, Haiderabad, the Indus Basin fossil failed rift, and the Thar rift areas), the highest surface thermal trends, ranging from 28°C to 31°C, can be observed. More moderate surface thermal trends, which range from 21°C to 26°C, are observed in the northern part of western Pakistan (Panjgur, Karan, and Chagai areas). Low surface trends are observed in the northern and the northwestern part of Pakistan (the Quetta, Chitral, Islamabad, Peshawar, and Gilgit areas).Zaigham NA (2010) [21] Interpolated thermal gradient data from about 60 exploratory wells, which shows the location of potential geothermal sources in the Indus Basin. The area between Karachi, Badin, east of Sibi, and west of Bahawalpur contains geothermal gradient anomalies that range from 4°C/100 m to 4.5°C/100 m. The higher geothermal gradients northeast of Nawabshah and Sui correspond to the faulted sections of the Indus basin. In Pakistan, 1426 exploration wells were drilled during the period from 1868 to 2016 over an area of 827,268 km^2^. About 450 of these wells are abandoned. Previous studies have shown that the focus of exploration activity shifted from the northern to the southern parts of Pakistan (Indus Basin) during the period from 1868 to 2016 [28, 29]. Moreover, aeromagnetic studies have been performed to locate geothermal sources of energy in the western parts of the Indus Basin [21] and have confirmed that the abandoned wells could be potential sources of electricity.

## Materials and methods

### Geology of the Indus Basin

The Lower Indus Basin is about 250km wide and bounded by the Thar Desert, which itself is bordered by Quaternary Hakra River flood-associated sediments. The Thar Desert lies on a structural platform and has a shallow granitic basement. To fully understand the area’s geology, a stratigraphic framework is needed [30]. The Chiltan Formation is a thick-bedded dark-colored limestone, and its texture varies from fine-grained, sub-lithography to oolitic and reefoid [31, 32]. The Cretaceous strata in Pakistan contain volcanic rocks with abducted masses of mélanges, ophiolites, and igneous intrusions making them high-temperature formations. The Cretaceous sediments of the southern Indus Basin include shale, sandstone, conglomerate, and limestone. One can see from the heterogeneous lithological characteristics of the Cretaceous formations that geological processes took place during which the subsurface temperatures were high. The Chiltan Formation extends to depths of 4500m and is in direct contact with the Cretaceous Sember Formation [33]. Zaigham NA (2000) [30] located more than 100 wells in the Indus Basin as deep as 4000 m, most of which are abandoned. The Chiltan Formation lies below the Upper Cretaceous Parh Formation. The thermal gradient measured at the Parh Formation is 3.0 to 3.39°C/100m on average, but at some points, such as at Giandari, it is abnormally high, i.e., 4.1°C/100m. Hence, the wells that are drilled into the Chiltan Formation, where the thermal gradient is more than 4°C/100m on average, and their use as geothermal reservoirs would be appropriate [30]. The Chiltan Formation is one of the failed Rift-associated tectonic regimes in the Indus Basin and is the location of the abandoned gas well used that is retrofitted into a geothermal reservoir make favorable for its development.

**Fig 1:**
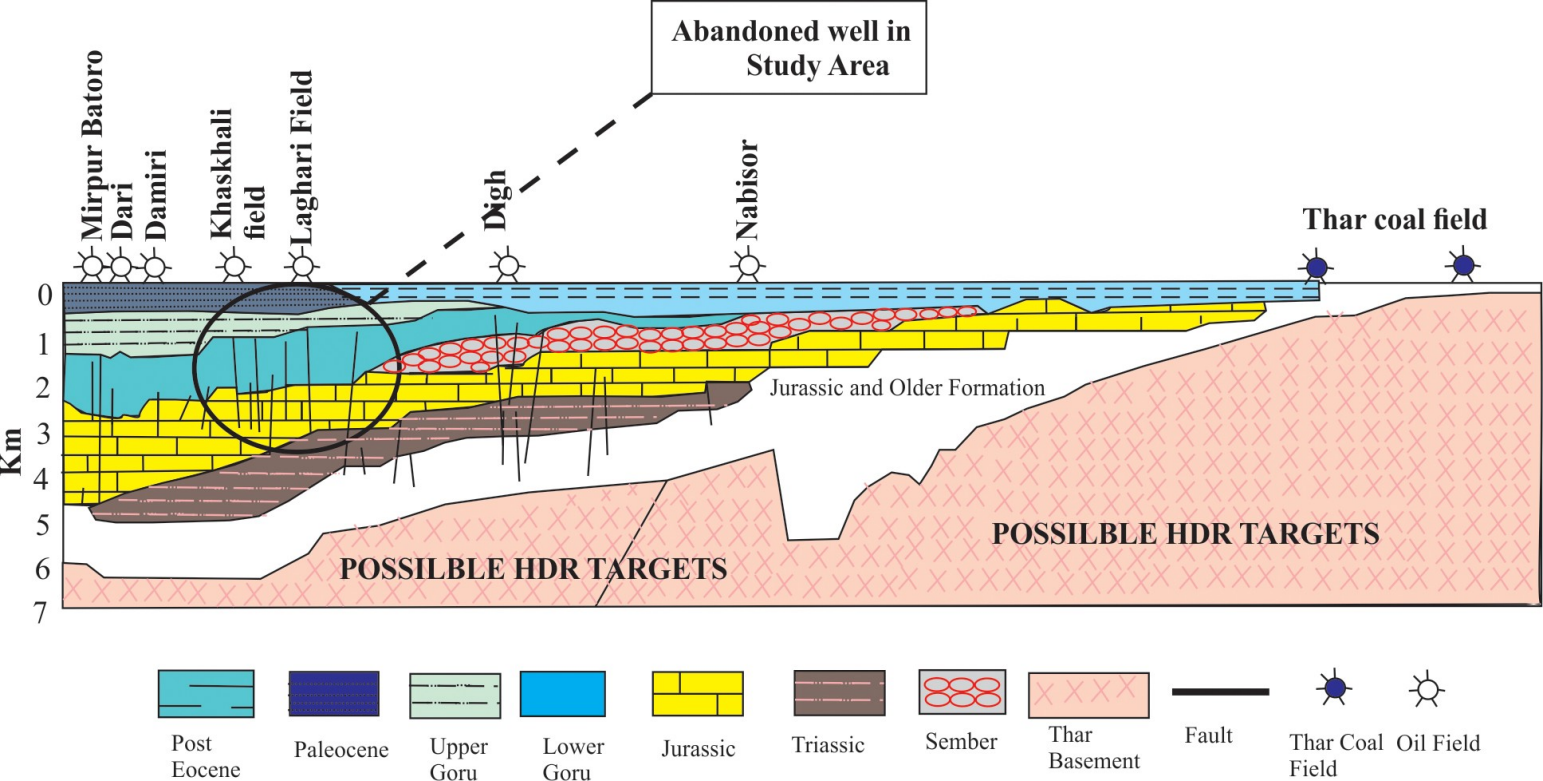
Geological cross-section of the Thar-rift in the southern part of the Indus Basin [21].

Seismic profile analysis shows that the stratigraphic sequences dip gently toward the northwest without major tectonic deformation. Fig 1 displays a schematic section that shows the sedimentary sequence decreasing in one direction and increasing in the opposite direction.

As discussed above, the rifted structures in the study area are favorable for the developing of geothermal reservoirs in the Indus Basin. From the available bottom hole temperature data, one sees that the thermal gradient increases from east to west in the L block within the Thar and Indus failed rift regions.

It is apparent that numerous geothermal resources are present in the study area and it appears that none of them has been developed in Pakistan. In the present study, a cost-effective method to provide a commercial and renewable alternative to Pakistan’s electricity generation by utilizing abandoned oil and gas wells for the extraction of geothermal energy is presented.

#### Case study

In the Thar failed Rift area, an abandoned gas well (SHAN-X1) was selected for the case study. SHAN-X1 is the westernmost well in the L block and is situated on the northwestern side of the Thar failed rift. In SHAN-X1, the geothermal gradient is 4.3°C/100 m due to a huge suite of underlying heat-generating granites at depths of less than 5 km.

**Fig 2:**
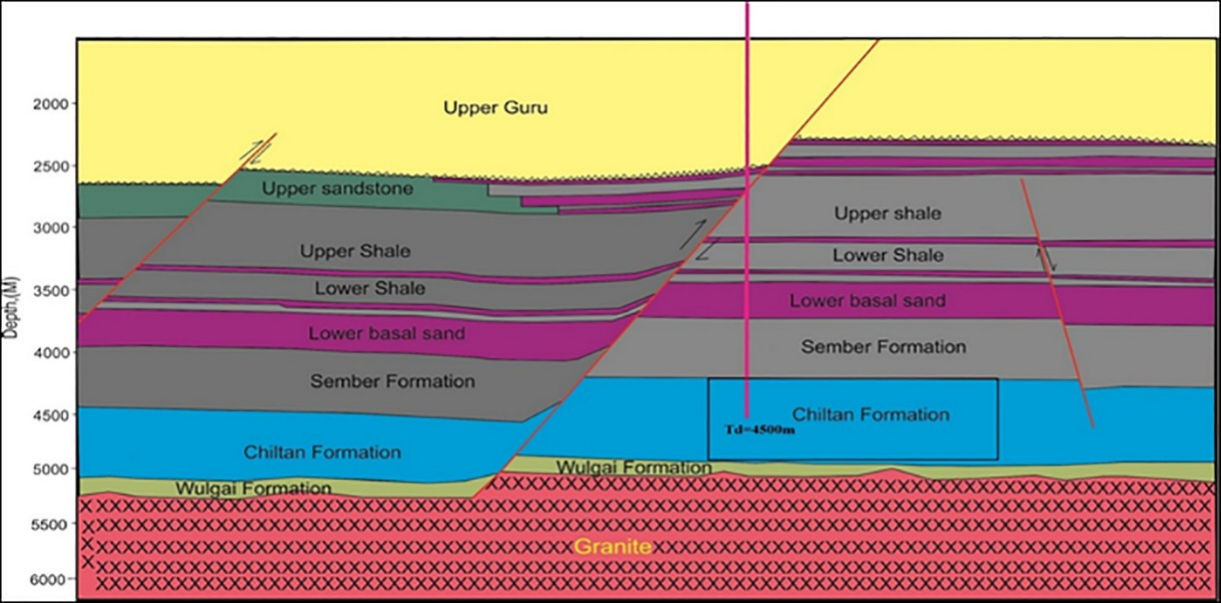
Structural cross-section of SHAN-X1. The rectangle in Chiltan Formation highlights the reservoir.

SHAN-X1 has a total depth (TD) of 4500m and is in the Chiltan Formation (Fig 2). The primary reason for drilling the SHAN-X1 well was to evaluate the hydrocarbon potential in the Chiltan limestone fractured reservoir. Wireline log analysis indicated that the reservoir has a gross column of 500 m with an average porosity of 10–14% and an average water saturation level of 85–90%. At 4500m a bottom hole temperature of 194°C was recorded. Within the Chiltan Formation, (only) gas was found in variable percentages ranging from 10–15%.

Due to the low quantity of hydrocarbons, exploiting SHAN-X1 for fossil fuel was uneconomical. However, the water saturation level of between 85–90% and 194°C bottom hole temperature make it a viable geothermal reservoir [34, 35]. SHAN-X1 was assumed to be an injection well. In the model, a second well was placed 200m from SHAN-X1 for extraction of the geothermal heat. Noteworthy, there are usually some corrosive elements present in the subsurface that may damage and corrode the equipment design and proper disposal. In case of the presence of such elements the equipment design and operational requirement will be considered accordingly and will not affect technicality of the presented concept of a geothermal system. Nevertheless, no traces of sour gases (H_2_S, CO_2_) and heavy resins were found in this area which makes the study reservoir more feasible for the presented concept. Hence, less attention is required for equipment design and operational requirement. But still massive research and evaluation are supposed to be conducted in the area to assure the possibility of potential challenges. A mathematical and numerical model was developed to study the thermal and hydraulic processes taking place in the fracture porous reservoir. Water seepage and heat transfer processes were also considered.

### Mathematical model

A three-dimensional geothermal numerical model was established to study the thermal recovery processes. By assuming local thermal non-equilibrium, the geothermal model uses energy equations to construct the temperature field of the existing fluid within a fractured rock matrix. The temperature field can then be used to describe the process of heat transfer from the actual rock-fluid during the heat recovery process.

#### Mass conservation equation

The mass balance equation describing fluid flow through a deformable porous media is given by Eq 1 [36, 37].

$$S\frac{\partial p}{\partial t}+\nabla \cdot u=-\frac{\partial e}{\partial t}+Q$$ (1)

In Eq 1, *S*, *P*, and *t* represent the constrained specific storage of the media, pressure, and time, respectively; *e* represents the volumetric strain, and *Q* represents the source-sink of the seepage process. As the fluid flow obeys Darcy’s Law, the water flow rate *u* can be calculated by:

$$u=-\frac{\kappa }{\mu }(\nabla p+\rho_{f}g\nabla z)$$ (2)

where *κ* represents the permeability of the saturated porous media; *μ* and *ρ*_*f*_ are dynamic viscosity and fluid density; *g* is gravitational acceleration; and the unit vector *z* represents the direction of gravity.

The mass balance equation for fractures is calculated using Eqs 3 and 4 [36, 38].

$$d_{f}S_{f}\frac{\partial p}{\partial t}+\nabla_{\tau }\cdot u_{f}=-d_{f}\frac{\partial e_{f}}{\partial t}+Q_{f}$$ (3)

$$u_{f}=-d_{f}\frac{\kappa_{f}}{\mu }\left(\nabla_{\tau }p+\rho_{f}g\nabla_{\tau }z\right)$$ (4)

where, *S*_*f*_, *κ*_*f*_, *d*_*f*_, and *e*_*f*_ denote the specific storage, permeability, thickness, and volumetric strain of the fractures, respectively; ∇_*τ*_ denotes the gradient operator; and *Q*_*f*_ represents the flow of fluid in the fractures and is given by $Q_{f}=-\frac{\kappa_{f}}{\mu }\frac{\partial p}{\partial n}$, where *n* represents the normal direction of the fracture surface.

#### Governing equation of the rock mass temperature field

Due to a decrease in porosity, the velocity of water in the rock matrix will be low; thus, the water temperature is assumed to be equivalent to the rock temperature.

$$C_{s}\rho_{s}\frac{\partial T_{s}}{\partial t}=\lambda_{s}\nabla^{2}T_{s}+W$$ (5)

where *ρ*_*s*_ is the density of the rock; *λ*_s_ represents the thermal conductivity of the rock matrix; C_s_ is the heat capacity of the rock; “*W*” represents the heat exchange in the reservoir; a negative sign means that heat is extracted from the rock, and a positive sign means that heat is absorbed by the fluid.

#### Governing equation of the fracture water temperature field

$$d_{f}\rho_{f}C_{f}\frac{\partial T_{f}}{\partial t}+d_{f}\rho_{f}C_{f}\cdot u_{f}\nabla_{\tau }T_{f}=d_{f}\nabla_{\tau }\cdot \left(\lambda_{f}\nabla_{\tau }T_{f}\right)+W_{f}$$ (6)

In Eq 6, *ρ*_*f*_, *C*_*f*_, and *λ*_*f*_ are the density, heat capacity, and thermal conductivity of water, respectively; *u*_*f*_ and *T*_*f*_ represent the water flow velocity and water temperature within the fractures, respectively; *W*_*f*_ represents the heat absorbed by the water from the matrix block on the fractured surface [39].

During the exchange of heat between the water and the rock matrix and fractures, it is assumed that water obeys Newton’s law of heat transfer. The flow of heat from the rock to the fracture fluid (water per unit area) is described by Eq 7 [40].

$$W=h(T_{s}-T_{f})$$ (7)

When the convection efficiency *h* is sufficiently large, the rock temperature and water temperature become equal at the fracture surface.

#### Fluid properties under high temperature and pressure

The coupling effect is related to the fluid properties, and in the deep geothermal reservoirs under high enough temperature and pressure conditions the fluid density *ρ*_*f*_ (water density), which can be defined as a function of temperature and pressure [40], becomes variable and satisfies:

$$1/\rho_{f}=3.086-0.899017{\left(4014.15-T\right)}^{0.147166}-0.39{\left(658.15-T\right)}^{-1.6}(p-225.5)+\delta$$ (8)

where *δ* is a function of water temperature *T* and pressure *p*; *δ* generally remains below 6% of 1/*ρ*_*f*_ and influences the dynamic viscosity *μ* = *υρ*_*f*_, where *υ* represent the kinematic viscosity of water. The kinematic viscosity is defined by Eq 9 [41].

$$\upsilon =\frac{0.01775}{1+0.033T_{f}+0.000221T_{f}^{2}}$$ (9)

The fluid properties are affected by the temperature and can influence the thermal and hydraulic coupling processes within a reservoir.

The validation of a numerical model for thermo-hydraulic analysis has been performed in the previous work [42].

### Thermal and hydraulic numerical model and simulation solution

**Fig 3:**
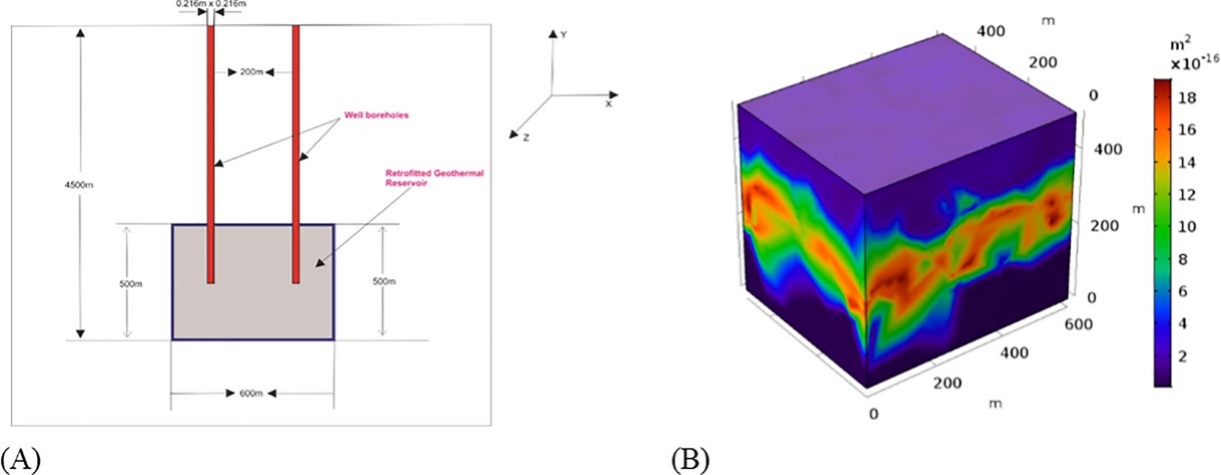
Schematic of the doublet well system considered. (A) Geometrical representation of doublet well system on the xy-plane (unit: m). (B) 3D sedimentary geothermal exploitation model (unit: m).

After applying the initial and boundary conditions, the thermal and hydraulic coupling analysis was carried out and a 3D computational model was constructed based on actual geological parameters. The retrofitted geothermal reservoir was 500m×600m×500m and was 4500m deep. x represents the horizontal direction, z the vertical direction, and y is perpendicular to x and z. The diagram is shown in Fig 3B represents the fractured zone assuming high permeability and a 200m to 250m depth and this part of the reservoir used as a geothermal reservoir. The injection well intersects the geothermal reservoir at x = 200, y = 250, and z = 250, and the production well intersects the reservoir at x = 400, y = 250, and z = 250. The reason for the intersection of injection and production well at this position is to keep the wellbores in the fractured area, with good reservoir properties. The wellbore radius is 216mm, and the horizontal section length is 200m as shown in Fig 3A. COMSOL Multiphysics was employed to solve the model numerically and the finite element method was used (Tetrahedra 564470, Triangles 16680, Edge elements 3660, Vertex elements 24).

#### Initial and boundary conditions

Based on the initial and boundary conditions, the heat production of the geothermal system was simulated using the proposed model and the thermal hydraulic coupling analysis was run for 50 years (time step is 1 day). The selected initial and boundary conditions are given below:

Seepage field: To ensure that water was circulated within the targeted reservoir, the pressure at the injection well was maintained at 25MPa, and the production pressure was 10MPa.

Thermal field: At the external thermal boundaries, the heat flux (Q_S_) was constant, whereas the surface temperature at the injection well was 30°C. The initial temperature in the reservoir was 194°C for both rock and water.

Tables 1 and 2 show the actual and adopted parameters used in the computational processes whereas the adopted data listed in Table 2 is based on engineering guesses.

**Table 1:** Actual geological parameters used for modeling.

Parameters	Symbol	Value
Density of rock	ρ_s_	2630 (kg/m^3^)
Heat capacity of rock	c_s_	850 (J/kg/K)
Heat flux	Q_s_	2000 (W/m^2^)
Porosity	ϕ	10–12 (%)
Average rock permeability	k_r_	1.0×10^−14^–2.0×10^−16^ (m^2^)
Reservoir length	L	600 (m)
Reservoir height	H	500 (m)
Reservoir temperature	T	194 (°C)

**Table 2:** Adopted parameters used for modeling [42].

Parameters	Symbol	Value
Density of fluid	ρ_f_	990 (kg/m^3^)
Thermal conductivity of the fluid	k_f_	0.6 (W/m/K)
Dynamic viscosity of the fluid	η	0.001 (Pa•s)
Thermal conductivity of rock	k_s_	3 (W/m/K)
Water production rate	q	49.55 (Kg/s)
Gravity acceleration	g_const_	9.8 (m/s^2^)
Convection coefficient	H	3000 (W/m^2^/K)

### Thermal recovery evaluation system

Evans K (2010) [43] proposed and evaluated the parameters used in the commercial exploitation of a thermal reservoir: (1) The minimum reservoir temperature is 190°C, and after 15–20 years of production, the reservoir temperature can drop less than 10% without reconstruction; (2) Water loss is less than 10% when the production rate is 100kg/s; (3) The volume of reservoir reconstruction is greater than 2×10^8^m^3^; and (4) The area of the effective heat exchange is greater than 2×10^6^m^2^. When the water injection rate is 50kg/s and the injection water temperature is 60°C, the commercial doublet well system generates electricity and heat at a rate of 3.5MW and 25MW, respectively [43]. Sanyal SK (2005) [44] suggested that the three most significant indicators required to assess a thermal reservoir’s performance are the thermal recovery rate, temperature production profiles, and net power generation profiles.

#### Thermal recovery runtime

Although geothermal energy is a renewable energy resource, the mean project runtime was 20 to 30 years. The temperature of a fractured reservoir in a geothermal system after thermal recovery drastically decreases and could take nearly 100 years to restore [45]. Therefore, it is essential to bind the geothermal energy exploitation to maintain sustainable development. Earlier studies have shown that the best time to stop thermal recovery is when the temperature decreases by 10°C [46] or when the temperature of the production well is depleted by 10% [47].

#### Average outlet temperature

The output temperature of the well is calculated using Eq 10,

$$T_{out}=\frac{\int_{L}u_{f}d_{f}T_{f}dL+\iint_{\Gamma }uT_{s}d\Gamma }{\int_{L}u_{f}d_{f}dL+\iint_{\Gamma }ud\Gamma }$$ (10)

where the outlet flow rate from fractures and the rock matrix to the production well are represented by *u*_*f*_ and *u*, respectively. The fracture width is represented by m; *T*_*f*_ and *T*_*s*_ represent the outlet water temperatures from the fractures and matrix rock, respectively; *L* represents the length integral of the output along with the fractures; and Γ represents the surface integral of the output along the matrix block [48].

#### Heat production

Heat production of the doublet well system is calculated by [49]:

$$W_{h}=q(h_{pro}-h_{inj})$$ (11)

where *h*_*inj*_ is the injection-specific enthalpy, *h*_*inj*_ = *C*_*p*_×*T*_*inj*_, *C*_*p*_ represent the specific heat capacity, *T*_*inj*_ is injection temperature; *h*_*pro*_ is the production-specific enthalpy, *h*_*pro*_ = *C*_*p*_×*T*_*pro*_,where *T*_*pro*_ represent the production temperature; and *q* is the total production rate.

After long-term exploitation, the heat transfer between the surrounding rock and the wellbore fluid can be ignored. The flow in the wellbore is essentially an iso-enthalpic process [49].

#### Energy efficiency

The system energy efficiency *η* is the ratio of the total production energy to the total consumed energy:

1. Total production energy
  Assuming all produced heat is consumed to generate electricity, then from the second law of thermodynamics, the useful work transformed is defined as:
  $$W_{e}=q(h_{pro}-h_{inj})(1-T_{o}/T_{pro})$$ (12)
  If the utilization efficiency coefficient of conversion from useful work to electric energy is 0.45 [44], then the equation for power generation is:
  $$W_{e}=0.45q(h_{pro}-h_{inj})(1-T_{o}/T_{pro})$$ (13)
  where *T*_*o*_ (absolute temperature) represents the reinjection temperature of injection well [44];
2. Total consumed energy
  The total consumed energy (*W*_*p*_) is primarily composed of energy consumed by the injection pump (*W*_*p*1_) plus the energy consumed by the suction pump (*W*_*p*2_). Ignoring the energy consumed by pipeline friction and internal fluid friction [50], the mathematical expressions for the total energy consumed are given by:
  $$W_{p1}=\int p_{inj}dV=q(P_{inj}-\rho gH_{1})/(\rho \eta_{p})$$ (14)
  $$W_{p2}=\int p_{pro}dV=q(\rho gH_{2}-P_{pro})/(\rho \eta_{p})$$ (15)
  $$W_{p}=W_{p1}+W_{p2}=\frac{q(P_{inj}-P_{pro})-\rho gq(H_{1}-H_{2})}{\rho \eta_{p}}$$ (16)
  where *dV* represents the volume of injected water per second; *H*_1_ and *H*_2_ represent the depth of the injection and production wells in meters, respectively; and *η*_*p*_ represents the pump efficiency;
3. System energy efficiency
  Based on heat production, the energy consumed *η*_*h*_ is given as:
  $$\eta_{h}=\frac{W_{h}}{W_{p}}=\frac{\rho \eta_{p}(h_{pro}-h_{inj})}{\left(P_{inj}-P_{pro})-\rho g(H_{1}-H_{2}\right)}$$ (17)
  Based on the production of electricity, the energy consumed (*η*_*e*_) is as follows:
  $$\eta_{e}=\frac{W_{e}}{W_{p}}=\frac{0.45\rho \eta_{p}(h_{pro}-h_{inj})(1-T_{o}/T_{pro})}{\left(P_{inj}-P_{pro})-\rho g(H_{1}-H_{2}\right)}$$ (18)

#### Rate of thermal recovery

1. Local thermal recovery rate

The local thermal recovery rate *γ*_*L*_ is defined as [51]:

$$\gamma_{L}(t)=\frac{T_{r}-T_{s}(t)}{T_{r}-T_{inj}}$$ (19)

where *T*_*r*_ and *T*_*s*_(*t*) represent the rock temperature at the initial time and at time *t*, respectively; and *T*_*inj*_ is the temperature of the injected fluid;

1. Overall thermal recovery rate

The ratio of thermal energy exploited by a geothermal system any time "*t*" to the total energy available in a thermal reservoir is the overall thermal recovery rate *γ* and is given by Eq 20 [51].

$$\gamma (t)=\frac{{∭}_{V}\gamma_{L}(t)dV}{{∭}_{V}dV}$$ (20)

## Results and discussion

### Sensitivity analysis of the outlet temperature

**Fig 4:**
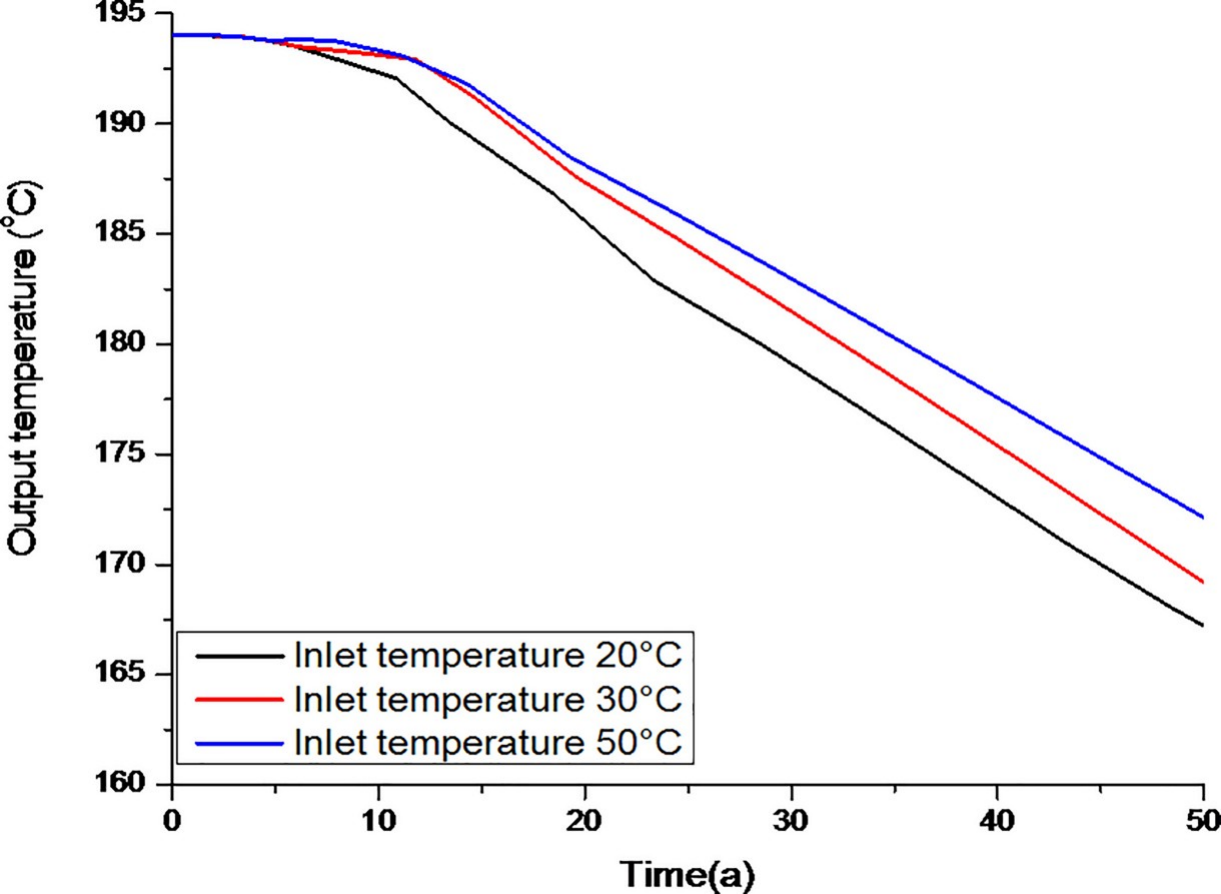
Evolution of the average outlet temperature assuming different inlet temperatures (unit: °C).

The life span and production performance of a retrofitted geothermal system are strongly dependent on the output temperature of the production well as mentioned in Eq 13. In a retrofitted geothermal reservoir, the lower the temperature of either the injected or the re-injected fluid, the higher the heat transfer into the reservoir, which results in higher production performance and longer lifespan. To evaluate the output temperature with respect to time, different injection temperatures were assumed, i.e., 20°C, 30°C, and 50°C (shown in Fig 4). During the first 12 years, conditions were stable. However, after 12 years, unstable conditions were observed due to the expansion of the lower temperature zone. The output temperature depended on the inlet temperature; the average temperature decreased 11–14% over the 50 year period, which significantly reduced the efficiency of the system. Too high an injection temperature can cause damage to the well assembly and bottom hole equipment. Even though high-grade metals are used to design the equipment, a high injection temperature over a long period weakens the bottom hole assembly. On the other hand, if the injection temperature is too low, heat flow into the subsurface thermal environment is insufficient to allow the thermal flow to reach the desired level.

**Fig 5:**
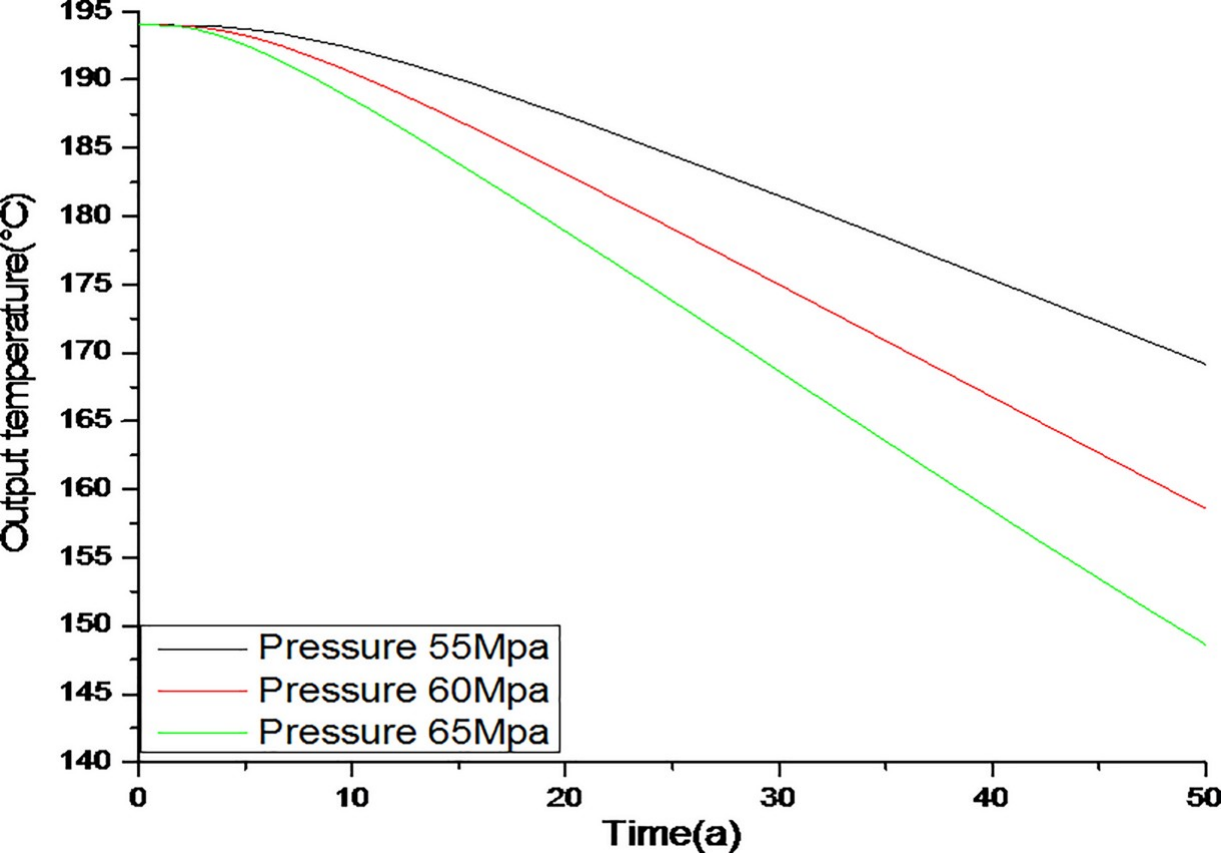
Evolution of the average outlet temperature with different inlet pressures (unit: MPa).

A graph of the average outlet temperature with respect to differences in the injection pressure is shown in Fig 5. The average outlet temperature was indeed influenced by the injection pressure. At a pressure of 25MPa, the outlet temperature remained stable during the first 12 years, but between 12 and 50 years the temperature decreased from 191°C to 167°C. When the injection pressure was 30MPa, the outlet temperature was stable during the first 9 years, but after that the output temperature decreased gradually from 191°C to 157°C. When the injection pressure was 35MPa, the average outlet temperature dropped rapidly to 136°C during the 50 year study period. Extremely high injection pressures can lead to an early thermal break between the injection and production wells due to earlier initiation of the convective process in an open loop geothermal system. Therefore, the injection pressure must not be too high.

**Fig 6:**
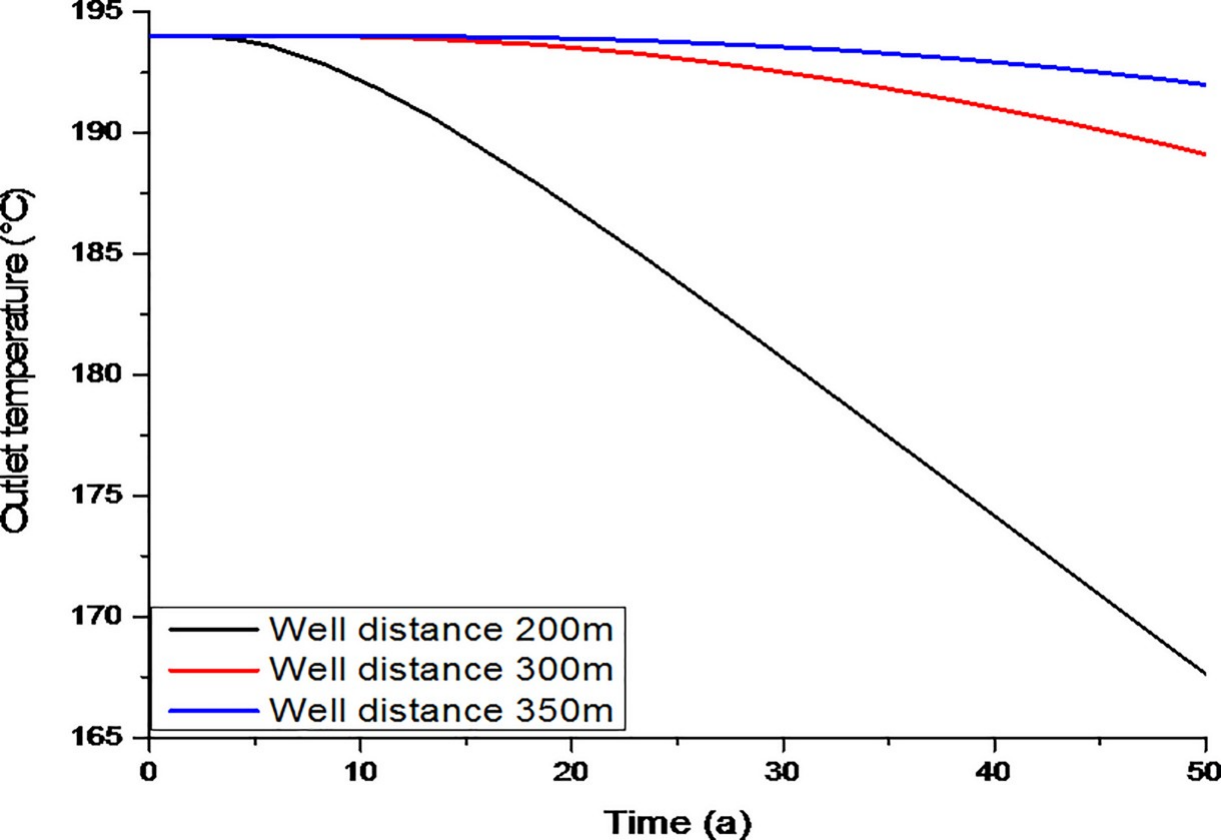
Evolution of the average outlet temperature with different distances (m) between the production and injection wells.

Fig 6 shows a graph of the average outlet temperature versus the distance between the injection and production wells (i.e., 200m, 300m, and 350m). The temperature of the production well varied over time with variation in the injection well distance from the production well. For well distances larger than 350m, the outlet temperature remained stable over the 50 year study period. However, when the distance between the injection and production wells decreased, the average outlet temperature decreased dramatically, which reduced the period in which the system was stable. The residence time and amount of heat exchange were greatly influenced by the distance between the two wells. A suitable distance between the injection and production wells is necessary to prevent a thermal breakthrough. If the distance between the injection and production wells is too large, water loss is likely, but the energy exchange between the rocks and fluid will be acceptable. An injection well too close to the production well will lead to an early thermal breakthrough due to a lower flux of heat between the rocks and the fluid. Thus, the distance between the injection and production wells plays a crucial role in the service life and performance of the system.

### Production temperature and thermal recovery lifetime

**Fig 7:**
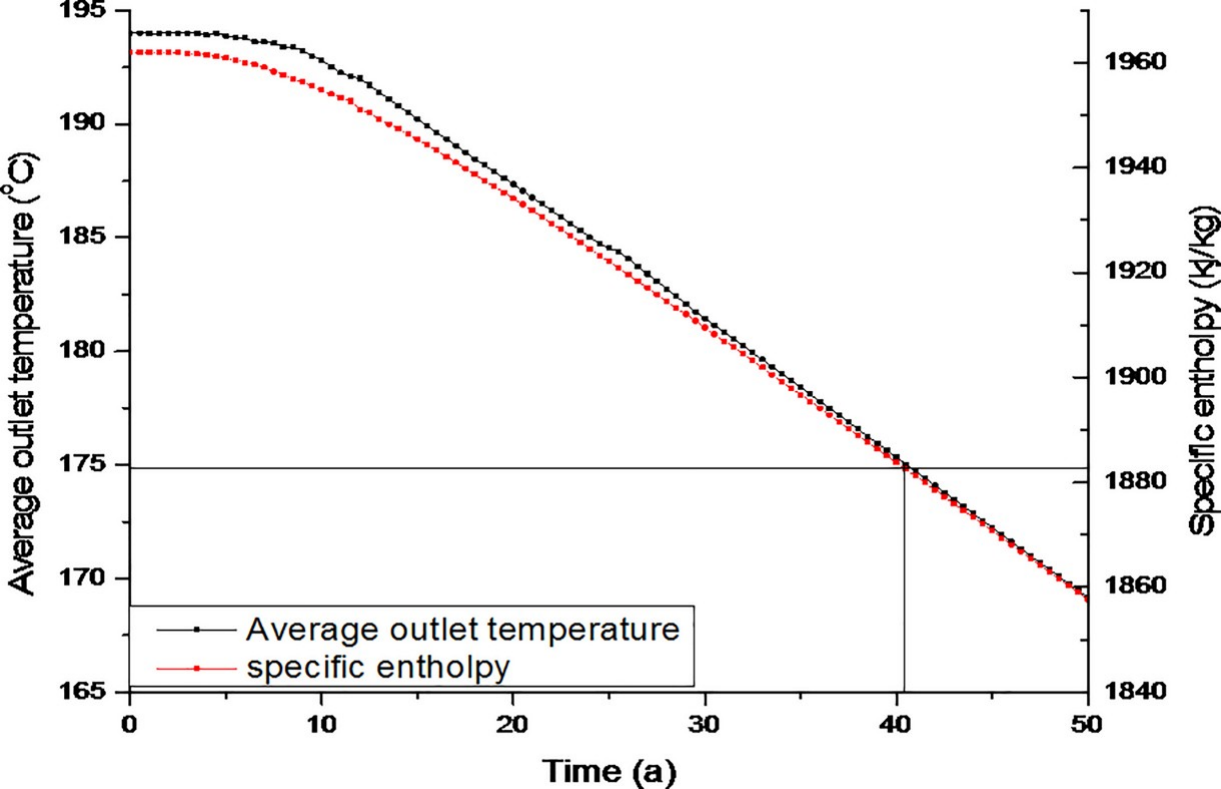
Evolution of the outlet temperature and specific enthalpy throughout the 50-year study period.

According to Eq 13 total production of energy strongly depends on the production temperature that represents the output temperature correlate with the energy harvested out of the system. Fig 7 shows the evolution of the average production temperature *T*_*out*_ and the production-specific enthalpy *h*_*out*_. The exploitation process can be divided into two phases: the steady phase and the decline phase. As shown in Fig 7, at the start of the operation the steady phase continued for approximately 10 years. During the first 10 years, the outlet temperature was maintained above 192°C, and the matching specific enthalpy remained stable, i.e., between 1962 kJ/kg and 1955 kJ/kg. During the period from 11–40 years, as shown in the graph, the outlet temperature of the water dropped from 192°C to 174.6°C and the specific enthalpy decreased from 1955 kJ/kg to 1881 kJ/kg. When the outlet temperature of the production well dropped to 174.6°C, i.e., when it dropped below 10% of the initial temperature, the process ended. The black lines in Fig 7 show that the life of this system was approximately 41 years, thus exceeding the commercial target of 15–20 years for thermal recovery runtimes.

#### Heat production and power generation

**Fig 8:**
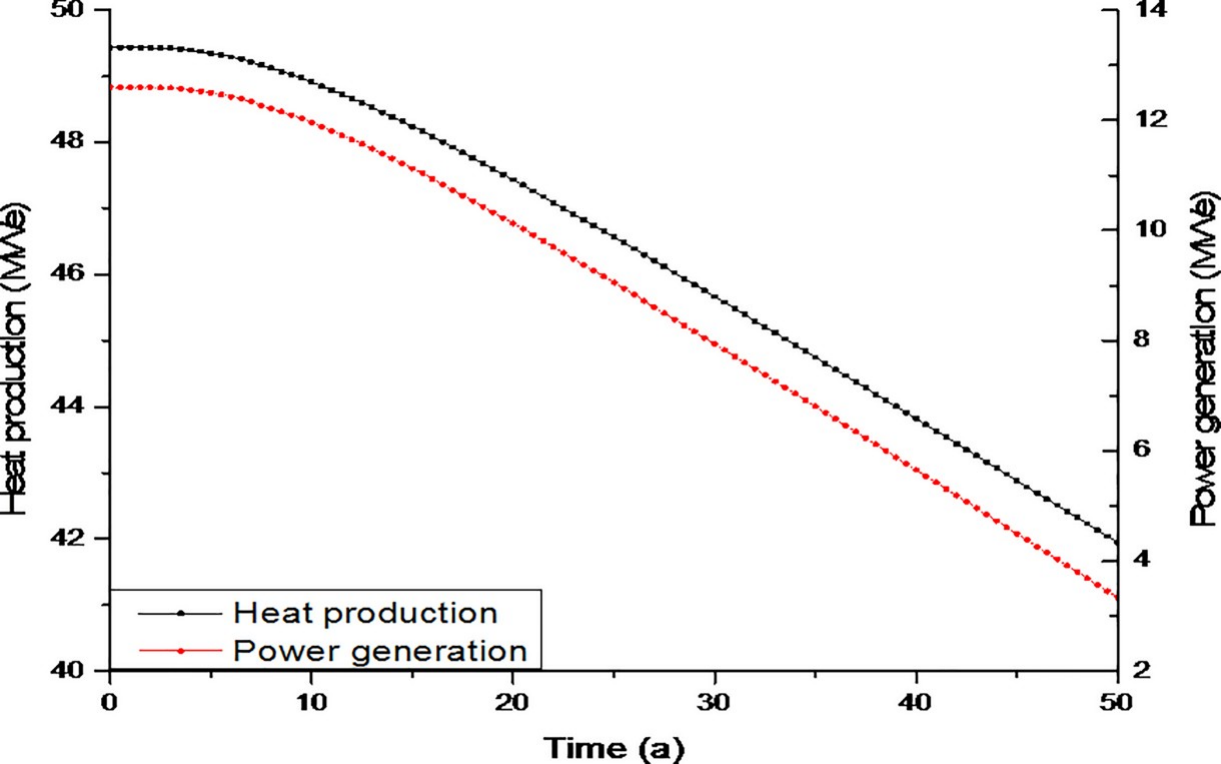
Evolution of heat production and power generation.

Fig 8 shows the evolution of heat production and power generation with respect to time. During the initial stage, heat production and power generation were at their maximum, after which they decreased gradually. The maximum heat production and power generation during the stable period were 49.4MWe and 12.6MWe, respectively. With the gradual decrease in temperature, the heat production decreased from 49.4MWe to 43.8MWe, and the power generation dropped from 12.6MWe to 5.6MWe after 40 years of exploitation. The commercialization target for the injection and production well system was 25MWe of heat and 3.5MWe of electricity [43]. Hence, the proposed system meets commercial requirements. Furthermore, as the Indus Basin is highly populated and has numerous power grid stations, the electricity generated from such a retrofitted geothermal system could be easily transferred to a nearby grid station. This process is not only less expensive than it would be if the grid stations were so numerous, but the transfer of power has the potential to serve large numbers of consumers.

#### Energy efficiency

**Fig 9:**
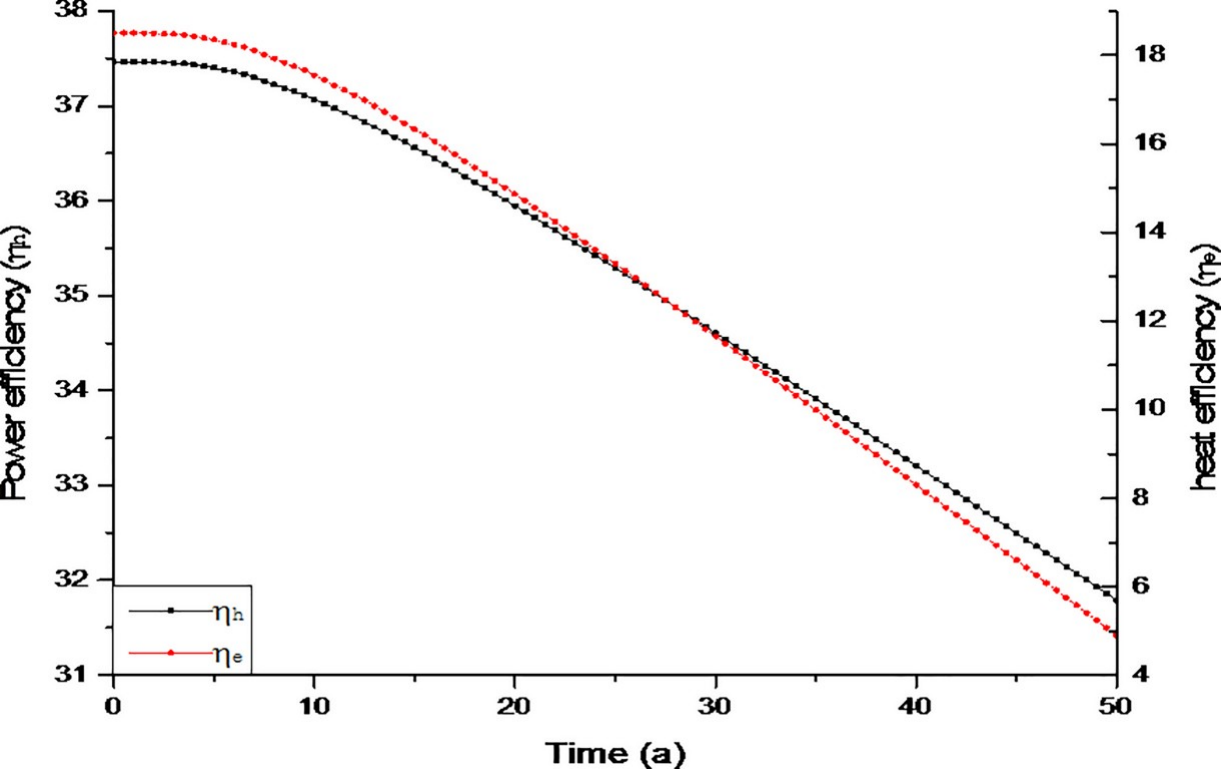
Evolution of energy efficiency *η*_*e*_ and *η*_*h*_.

Fig 9 shows the evolution of energy efficiency (*η*_*e*_) and heat efficiency (*η*_*h*_). The energy efficiency based on heat production and power generation declined with time. After 50 years, *η*_*e*_ decreased from 18.5 to 4.9 and *η*_*h*_ fell from 37.5 to 32.8. For economically viable exploitation, the cost of drilling, production and hydraulic fracturing must not exceed the value of the produced geothermal energy measured under the parametric evaluation of energy efficiency *η*_*e*_ and heat efficiency *η*_*h*_ [44]. Among these costs, the drilling is the highest cost engineering component and account for about 44% of the total project investment [52, 53]. However, by reusing the abandoned oil wells the drilling cost can be eliminated significantly, which makes the project more feasible and economical. Nevertheless, the outcomes of the current project are dependent on flash steam power plants, which operates on the temperature of hydrothermal fluids above 180°C [54]. Recent advancements in geothermal power generation technology like binary cycle plant the operational requirements for temperature limit can be reduced to 85°C -175°C in order to improvise the energy efficiency of the system.

#### Rate of heat recovery

**Fig 10:**
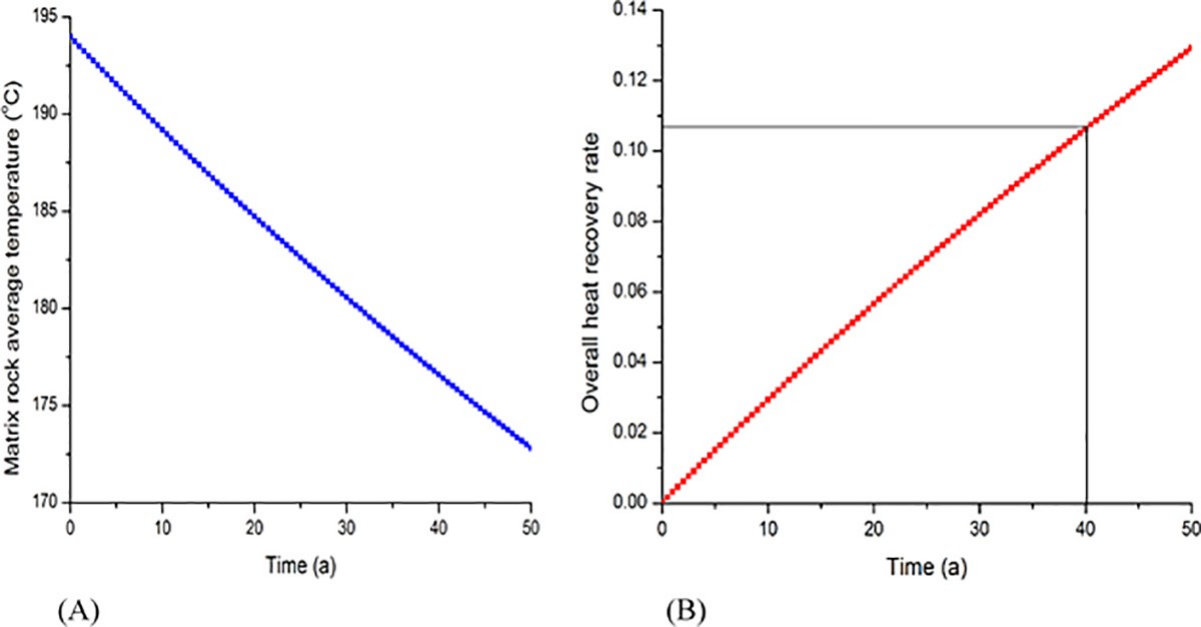
Evolution of matrix rock average temperature and overall heat recovery rate. (A) Evolution of matrix rock average temperature with respect to time. (B) Evolution of overall heat recovery rate with respect to time.

Fig 10A, Fig 10B show the average rock temperature and the thermal recovery rate in the reservoir. As one can see, these quantities are linear with respect to time. This linearity is due to the constant heat supply due to the system’s stable temperature (i.e., it remained within the maximum and minimum values). As the temperature approached these maximum/minimum values, the growth rate of the thermal energy decreased with time. However, at 40 years (as shown in Fig 10B), the thermal energy extraction dropped to 11%; thus, a large amount of thermal energy remained in the reservoir and optimized extraction methods were needed.

## Conclusions

In this paper, the heat transfer and flow regime of an injection-production well pair is modeled by first studying the geology of the Indus Basin at a location where oil and gas production has occurred. An abandoned gas reservoir is used to provide the geological parameters that are needed to construct a geothermal reservoir model. Using local thermal non-equilibrium theory, a thermal and hydraulic mathematical model and 3D numerical model were established to evaluate the extraction of heat from a retrofitted geothermal system. The following conclusions were made:

1. The lifespan of the 3D doublet well retrofitted geothermal system model is about 40 years, exceeding the commercial target and thermal recovery runtime of 15–20 years. Sensitivity analyses were performed to examine the effects of model parameters on the average production temperature of the retrofitted geothermal system. The average production temperature is sensitive to the injection temperature, injection pressure and distance between the injection well and production well.
2. Thermal recovery performance of the retrofitted geothermal system can be improved by optimization of the flow field distribution within a reservoir. Heat production and power generation were at first stable, but over time the stability decreased; this situation met the commercial requirements for a retrofitted geothermal system. On the other hand, energy efficiency based on Flash steam power plants is still low.
3. The substantial number of abandoned wells in the Indus basin presents a chance for the development of low-cost geothermal energy via a retrofitted geothermal system. The data from abandoned oil and gas wells associated with tectonically active areas can be used not only to minimize the time and exploration cost for retrofitted geothermal system development, but also to recover the economic losses incurred by abandoned/dry wells by repurposing them.
4. Although the overall heat recovery is not large enough, the production conditions, thermal reservoir construction design, and selection of an appropriate well configuration can be chosen to optimize the overall heat recovery. However, the research domain of future study can be expanded by improvising the technical factors that are production conditions, thermal reservoir construction design, and selection of an appropriate well configuration.

## Supporting information

S1 File Injection temperature at 20deg, Outlet temperature with respect to time. (TXT)

S2 File Injection temperature at 30deg, Outlet temperature with respect to time. (TXT)

S3 File Injection temperature at 50deg, Outlet temperature with respect to time. (TXT)

S4 File Injection pressure at 55Mpa, Outlet temperature with respect to time. (TXT)

S5 File Injection pressure at 60Mpa, Outlet temperature with respect to time. (TXT)

S6 File Injection pressure at 65Mpa, Outlet temperature with respect to time. (TXT)

S7 File Well distance 200m, Outlet temperature with respect to time. (TXT)

S8 File Well distance 300m, Outlet temperature with respect to time. (TXT)

S9 File Well distance 350m, Outlet temperature with respect to time. (TXT)

S10 File Mass flow rate with respect to time. (TXT)

S11 File Average outlet temperature and specific enthalpy with respect to time. (TXT)

S12 File Heat production and power generation with respect to time. (TXT)

S13 File Heat efficiency and power efficiency with respect to time. (TXT)

S14 File Average matrix temperature with respect to time. (TXT)

S15 File Overall thermal recovery with respect to time. (TXT)

## Nomenclature

Below are the definitions of the parameters used in this manuscript:

p: hydraulic pressure (Pa)
T_s_: rock temperature (K)
T_f_: water temperature in fracture (K
t: Time (s)
μ: dynamic viscosity of fluid (Pa.s)
u: water flux (m/s)
k_r_: permeability of rock (m^2^)
k_f_: permeability of fracture (m^2^)
S: storage coefficient of rock mass (1/Pa)
S_f_: storage coefficient of fracture (1/Pa)
Q: source/sink term of seepage process (1/s)
Q_f_: exchange of flow between rock mass and fracture surface (m/s)
d_f_: width of fracture (m)
ρ_s_: density of rock (kg/m^3^)
ρ_f_: density of water (kg/m^3^)
λ_s_: thermal conductivity of rock mass (W/m/K)
λ_f_: thermal conductivity of water (W/m/K)
C_s_: heat capacity of rock block (J/kg/K)
C_f_: heat capacity of water (J/kg/K)
W: source of heat (W/m^3^)
W_f_: absorption of heat from rock matrix block to fracture surface (W/m^2^)
u_f_: water flow velocity in fracture (m/s)
h: convection coefficient (W/m^2^/K)
T: absolute temperature (K)
u: kinematic viscosity coefficient
L: integral length of the output along fracture
T: surface integral of the output along the matrix rock block
h_inj_: injection specific enthalpy (kJ/kg)
h_pro_: production specific enthalpy (kJ/kg)
q: production rate (kg/s)
W_h_: heat production (MWe)
W_e_: power generation (MWe)
W_p1_: energy consumed by injection pump (MWe)
W_p2_: energy consumed by suction pump (MWe)
W_p_: overall energy consumption (MWe)
T_0_: reinjection temperature of inlet well (K)
η_p_: efficiency of pump
dV: volume of injected water per second (m^3^/s)
H_1_: depth of inlet well (m)
H_2_: depth of outlet well (m)
η_h_: energy consumed based on heat production
η_e_: Energy consumed based on power generation
T_inj_: temperature of inlet fluid (K)
γ_L_: local heat recovery rate
γ: overall heat recovery rate
T_r_: rock mass temperature at initial time (K)
T_s_(t): rock mass temperature at time t (K)
